# Hot Deformation Behavior and Processing Maps of ZnSnO_3_/Cu Composites

**DOI:** 10.3390/ma15207402

**Published:** 2022-10-21

**Authors:** Wei-Jian Li, Zi-Yao Chen, Xiao-Peng Tang, Wen-Zhu Shao, Liang Zhen

**Affiliations:** 1 College of Nuclear Equipment and Nuclear Engineering, Yantai University, Yantai 264005, China; 2 School of Materials Science and Engineering, Harbin Institute of Technology, Harbin 150001, China; 3 National Key Laboratory of Precision Hot Processing of Metals, Harbin Institute of Technology, Harbin 150001, China

**Keywords:** ZnSnO_3_/Cu composites, ternary oxides, hot deformation behavior, processing maps

## Abstract

In this work, we designed ternary ZnSnO_3_ particle-reinforced Cu matrix composites and evaluated the hot deformation behavior of ZnSnO_3_/Cu composites. The hot deformation characteristics of typical dynamic recrystallization were probed by the resulting true stress–strain curves of ZnSnO_3_/Cu composites. The influences of deformation conditions, including temperatures (650–850 °C) and strain rates (0.01–5 s^−1^), on the flow stress of the designed composites were investigated. This revealed that the peak stress increased with the increasing of strain rate and decreasing of temperature. Additionally, the activation energy was calculated to be 237.05 kJ/mol and followed by yielding a constitutive equation for low-stress ZnSnO_3_/Cu composites. The processing maps established by dynamic materials model theory indicated that the designed composites possessed excellent hot workability, and then the processing parameters (790–850 °C and 0.01–0.04 s^−1^) of the ZnSnO_3_/Cu composites were determined for practical industrial production. Our work discloses the deformation behavior of ZnSnO_3_/Cu matrix composites and extends the rational process design for ternary ceramic/metal materials with excellent hot workability.

## 1. Introduction

Benefited by remarkable electrical and thermal properties, copper has been widely used in the electronic power, machinery manufacturing, chemistry, and marine industries [1,2,3]. However, the low intrinsic strength of copper has become a major obstacle for its further application with the development of the electronic and electrical industries [4,5]. To address this issue, numerous reinforcement particles, including oxides, carbides, borides, and nitrides, were introduced to a pure Cu matrix yielding excellent comprehensive performance. For example, Al_2_O_3_ particles, as the reinforcement phase, have been commonly introduced to the copper matrix by various methods, such as internal oxidation and in situ synthesis. These dispersed Al_2_O_3_ particles could improve the threshold stress of dislocation glide and strengthen the composites by inducing extra dislocations around Al_2_O_3_ phases [6,7]. Vidyuk et al. synthesized the TiC/Cu composites with Cu-Ti-C by in-situ synthesis, achieving an excellent combination of ultimate compressive strength and electrical conductivity [8]. Yu. et al. produced 20 vol% TiB_2_/Cu matrix composites by exothermic dispersion synthesis with a Cu-Ti-B system, and the strengthening phase TiB_2_, as a reaction product, improved the ultimate tensile strength (318 MPa) of the materials over pure copper by 45% [9].

These binary ceramic particles can enhance the strength of Cu composites without severe deterioration of electrical conductivity. However, low plasticity and poor machinability notably arises in these composites. Under conditions of hot processing, such as rolling and hot extrusion, cracks are especially prone to initiate and propagate along the interface between the reinforcement phases with Cu matrix [10,11,12], leading to the damage and failure of the composite materials. Thus, devising a method to avoid the weak adhesion of phase interface and enhance plasticity has generated considerable interest. In this regard, extensive research has been dedicated to exploring the compression characteristics of particle-reinforced Cu-based composites to optimize processing parameters for practical industrial applications, including deformation temperature, strain rate, and deformation amount.

Abundant research results have been presented on the thermal deformation behavior of Cu materials [13,14,15]; however, for most composites that only combine ceramic particles (TiC [16], Al_2_O_3_ [17], WC [18], etc.) and copper matrix, achieving excellent plasticity and machinability is still a major challenge, and this considerably hinders the application of these composites. In comparison with simple binary ceramics, multication ceramics (La_2_NiO_4_, Zn_2_SnO_4_, Ti_3_AlC_2_, etc.) have garnered significant attention due to their elevated temperature strength, electrical conductivity, thermal shock resistance, and remarkable metal compatibilities [19,20,21]. Benefited by these merits, multication ceramics have been considered as one of the most promising reinforcement phases for a new type of Cu-based composites [22,23,24,25]. However, limited information is available on the thermal deformation behavior of multication ceramics/copper matrix composites, which significantly restricts the practical industrial production and application of these composites.

ZnSnO_3_, as a ZnO-SnO_2_ ternary compound, has more freedom to tune the material property of Cu-based composites compared with single binary ZnO or SnO_2_. ZnSnO_3_/Cu composites with excellent electrical conductivity, strength, and electric arc erosion resistance have been successfully applied in low-voltage electrical apparatuses, including alternating current contactors, delays, and breakers. Still, the hot deformation behavior of ZnSnO_3_/Cu composites remains to be further evaluated in order to satisfy the demands of industrial production.

In this work, cylindrical ZnSnO_3_/Cu composites were prepared via powder metallurgy, and the hot deformation behavior of ZnSnO_3_/Cu composites was investigated. The influences of various deformation conditions on the flow characteristics were evaluated, and the microstructures were characterized in order to illustrate the deformation mechanisms. Additionally, constitutive equations relating to the thermal deformation activation energy and *Z* parameter have been found. Processing maps have been depicted employing the dynamic material model and have been applied to rationally select hot working parameters for the industrial production of ternary oxide-reinforced copper matrix composites.

## 2. Experimental

**Figure 1:**
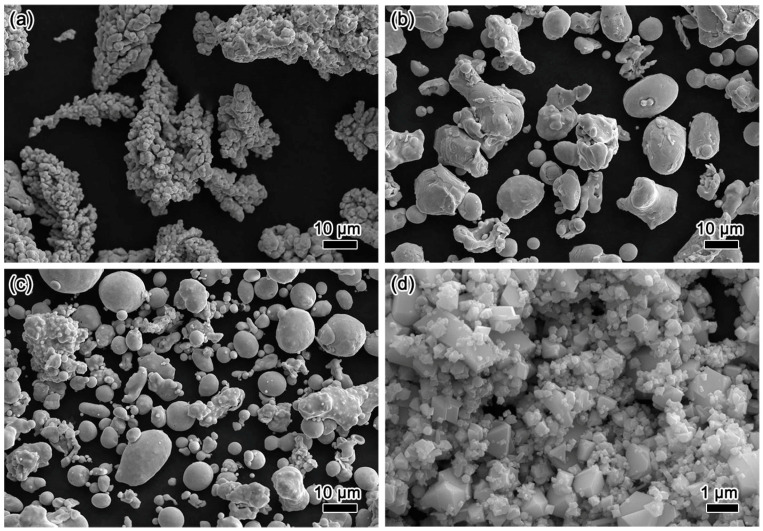
The morphology of electrolytic Cu powder (**a**), atomizing CuZr alloy powder (**b**), atomizing CuLa alloy powder (**c**), and ZnSnO_3_ powder (**d**).

The proportions of Zr, La, and ZnSnO_3_ in the prepared ZnSnO_3_/Cu composite were 0.15 wt.%, 0.005 wt.%, and 2 wt.%, respectively, and the remainder was Cu. The morphologies of the raw materials are shown in Figure 1. In this figure, the electrolytic Cu powders exhibit a dendritic morphology with size of 20–40 μm (Figure 1a). The atomizing CuZr alloy (Zr: 0.56 wt.%) and CuLa alloy (La: 0.35 wt.%) display quasi-spherical morphology and possess the size of 5–20 μm, as illustrated in Figure 1b,c, respectively. The morphologies of ZnSnO_3_ particles with a size of 0.2–1 μm are shown in Figure 1d.

**Figure 2:**
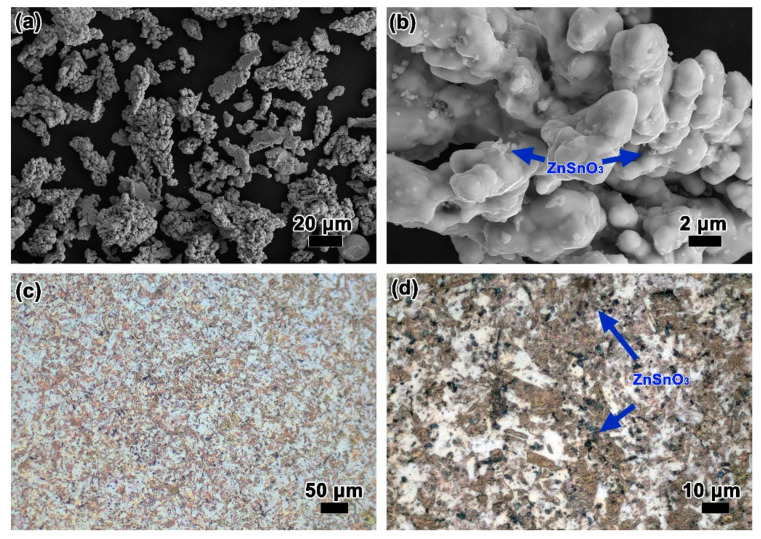
(**a**,**b**) The morphology of the mixed powders. (**c**,**d**) Microscopic structure of the ZnSnO_3_/Cu composites.

Three kinds of stainless steel balls with diameters of 3, 5, and 10 mm were used as the ball mill medium, and the weight ratio was 2:2:1. The stainless steel balls and the above raw materials with a weight ratio of 1:1 were mixed in a powder mixer (5 L) for 4 h at 80 revolutions per minute. The mixed powders were obtained after drying at 50 °C for 2 h. Figure 2a,b demonstrates that the ZnSnO_3_ particles can disperse uniformly in the mixed powders and especially in the middle of Cu branches. Next, the mixed powders were compressed into φ 8 × 12 mm cylindrical specimens under a pressure of 400 MPa. Afterwards, the specimens were sintered at 927 °C in Ar atmosphere for 4 h. After furnace cooling to room temperature, the samples were re-compressed at 700 MPa to increase the density of the specimens. Finally, the composites were annealed at 500 °C in Ar atmosphere for 2 h. The microscopic structures in Figure 2c,d show that the obtained ZnSnO_3_/Cu composite is uniform and dense in microstructure. The obtained samples were used for hot compressive tests.

The hot compressive testing was carried out employing a Gleeble-1500D thermal simulation test machine (Dynamic systems Inc., Anaheim, CA, USA). Four samples were tested under each deformation condition in order to guarantee the accuracy of the results. Graphite sheet was installed between the cylinder specimen and the pressure head to provide lubrication. After maintaining the target temperature for 2 min under vacuum conditions, the samples were compressed in different $\;\dot{\varepsilon }\;$ (0.01–5 s^−1^) with total deformation of 70%, and the true stress–strain curves were yielded. Hot processing maps were depicted based upon the DMM model [18]. All the quenched specimens were etched using a mixed reagent of ferric chloride (5 g), ethanol (85 mL), and hydrochloric acid (10 mL).

The morphologies of the raw materials and mixed powders were observed with scanning electron microscope (SEM, FEI Quanta 200F, Waltham, MA, USA). The metallographic microscope (Axiovert 40 MAT, Zeiss, Oberkochen, Germany) was used to observe the microstructures of ZnSnO_3_/Cu composites before and after hot deformation.

## 3. Results and Discussion

### 3.1. Flow Stress Behavior

**Figure 3:**
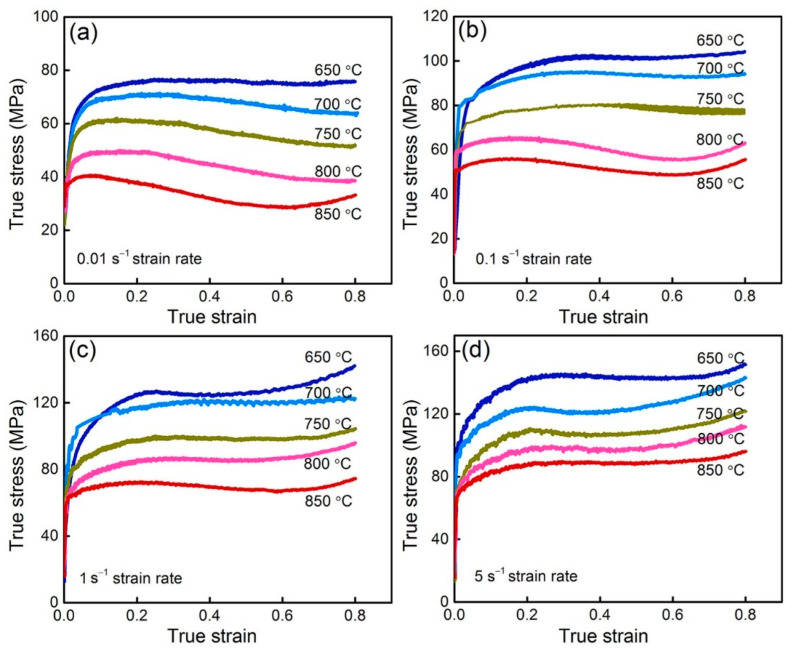
Relationship between the true stress and strain of the ZnSnO_3_/Cu composites at 650–850 °C and strain rates: (**a**) 0.01 s^−1^, (**b**) 0.1 s^−1^, (**c**) 1 s^−1^, and (**d**) 5 s^−1^.

Figure 3 depicts the relationship between the true stress and strain of ZnSnO_3_/Cu composites. Notably, a common feature can be observed in that, in the early deformation stage, the flow stress increases linearly and sharply with the increase of strain. This generally results from work-hardening owing to the dislocation generation and multiplication. Once the deformation degree reaches critical strain, dynamic recrystallization occurs, yielding a rise in the dislocation density. However, the work-hardening still acts as the dominant factor and leads to a slowing down of the rate of stress increase [26]. Upon further increasing strain, the dynamic recrystallization turns into a primary softening mechanism, weakening the flow stress. When these two enter a steady state of dynamic equilibrium, the flow stress remains steady in spite of the increase of strain [27,28]. These tests have been repeated several times, and the same tendencies were displayed in each testing. Similar phenomena also occurred in Cu alloys with low stacking fault, wherein the dislocation glide was restricted by the dislocation network and nodes, and the opposite sign dislocation failed to be offset by the dislocation climb or cross-slip [16,29,30,31]. In this case, the dynamic recovery was restricted. Thus, the softening mechanism of Cu-based materials during thermal deformation is mainly dynamic recrystallization.

Additionally, when the strain rate is determined, increasing the deformation temperature results in a decrease of the flow stress, which is ascribed to the thermal activation and the atomic thermal motion. The increased thermal activation accelerates the nucleation of dynamic recrystallization and enhances the driving force of growth of the recrystallized grains. Consequently, the softening action induced by the dynamic recrystallization of the composites is enhanced, decreasing the flow stress during deformation. The heat, on the other hand, accelerates the thermal motion of atoms, which reduces the critical shear stress for initiating dislocation sliding, and is followed by dislocation climbing and cross-slipping to reduce the flow stress. Additionally in this figure, once the temperature is constant, the increase to the strain rate obviously improves the flow stress due to the synergistic effects from work-hardening and recrystallization softening. As the strain rate increases, the speed of dislocation motion also increased. Thus, the critical shear stress for material deformation increased, causing an improvement of the flow stress. The rise of the strain rate shortens the deformation time of the composites, leading to the reduction of the dynamic recrystallization rate and growth rate. Thus, the reduction of the softening effect increases the flow stress of ZnSnO_3_/Cu composites. However, at a higher strain rate (1–5 s^−1^), the strain of the designed ZnSnO_3_/Cu composites can reach 0.8 due to larger quantity of slip systems in face-centered cubic copper, which is consistent with the results of previous studies [13,32].

### 3.2. Microstructure Analysis

**Figure 4:**
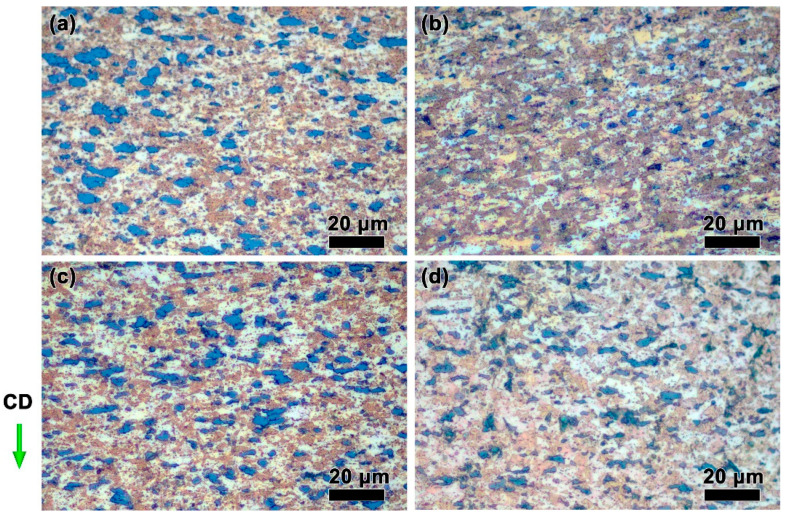
Microstructures of the ZnSnO_3_/Cu composites at 0.01 s^−1^ strain rates with different temperatures: (**a**) 650 °C, (**b**) 700 °C, (**c**) 750 °C, and (**d**) 800 °C. CD presents the compression direction of the sample.

Figure 4 shows the structures of the ZnSnO_3_/Cu composites deformed at a relatively lower strain rate (0.01 s^−1^) with different temperatures. Under relatively low deformation temperatures (Figure 4a,b), ZnSnO_3_ particles in black color tend to regularly array along the vertical axis to the compression direction (CD) in the Cu matrix. When increasing the deformation temperature, the characteristic regular arrangement of the second phase disappears, yielding a disordered state of the arrangement of ZnSnO_3_ particles at 800 °C (Figure 4d). This change highly relies upon the increased atomic thermal motion at high temperatures, and in this case, the motion of the second phases receives a corresponding rise and moves in parallel to the hot pressing, resulting in a disordered distribution of the ZnSnO_3_ particles. Comparing Figure 4a,b, the grains deformed at 800 °C (Figure 4d) obviously grow in size, which is ascribed to the enhanced driving force [33].

**Figure 5:**
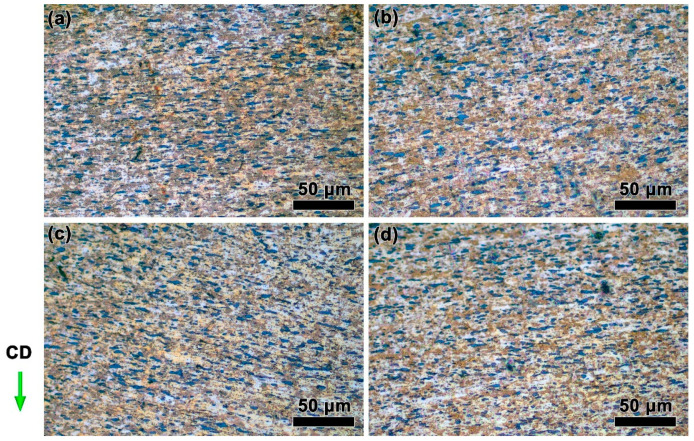
Microstructures of the ZnSnO_3_/Cu composites at 1 s^−1^ strain rates under different temperatures: (**a**) 650 °C, (**b**) 700 °C, (**c**) 750 °C, and (**d**) 800 °C. CD presents the compression direction of the sample.

Figure 5 shows the structures at a high strain rate (1 s^−1^) under different temperatures. It demonstrates that the second phase is regularly distributed along the vertical axis in the direction of deformation in the Cu matrix, even when the temperature increases to 800 °C. This can be explained by the shortened time for the movement of the second phases at a high strain rate, and the characteristic of regular arrangement of second phase is still present in the Cu matrix after quenching. Notably, a high strain rate is beneficial to the formation of fine recrystallized grains. Additionally, it is generally accepted that the grain size changes are accompanied by texture evolution. <001> texture, as the typical texture in copper, were reported to exist at low temperature and low strain rate [34]. As the temperature raised, the polar density of <001> texture was also observed to increase, which was ascribed to the accelerated recrystallization. It can be deduced from Figure 5 that the texture of the ZnSnO_3_/Cu composites was primarily of <001> texture, and the density of <001> texture could increase as the temperature and strain rate increased.

### 3.3. Constitutive Analysis

During deformation, the constitutive relationship between flow stress, strain rate, and temperature can be described by the Zener–Hollomon *Z* parameter, which can be written as [35,36,37,38]:

(1) $$\;\dot{\varepsilon }\;=A_{1}\sigma^{n^{1}}\exp\left(-\frac{Q}{RT}\right)$$

(2) $$\;\dot{\varepsilon }\;=A_{2}\exp\left(\beta \sigma \right)\exp\left(-\frac{Q}{RT}\right)$$

(3) $$\begin{array}{c} \;\dot{\varepsilon }\;=A{\left[\sin h\left(\alpha \sigma \right)\right]}^{n}\exp\left(-\frac{Q}{RT}\right) \end{array}$$

(4) $$\begin{array}{c} Z=\;\dot{\varepsilon }\exp\;\left(-\frac{Q}{RT}\right) \end{array}$$

Here, $\;\dot{\varepsilon }\;$ (s^−1^) denotes the strain rate, *σ* (MPa) is the flow stress for a certain strain, and *Q* (kJ mol^−1^) is the activation energy of DRX. *A* (s^−1^), *A*_1_ (s^−1^), *A*_2_ (s^−1^), *n*_1_, *n*, *β* (MPa^−1^), and *α* (MPa^−1^) (*α = β*/*n*_1_) are the material constants. Note that Equation (1) with exponential form is applicable to describe low stress deformation, and Equation (2) is adopted to describe high stress deformation. The hyperbolic sine relation [Equation (3)] is used to correct a wide range of stress deformation by introducing the Arrenius relation. It is assumed that ZnSnO_3_/Cu composites satisfy the above equations in the process of thermal deformation. Taking natural logarithms on both sides of Equations (1) and (2) yields:

(5) $$\begin{array}{c} \ln\;\dot{\varepsilon }\;={\ln A}_{1}{+n}_{1}\ln\sigma -\frac{Q}{RT} \end{array}$$

(6) $$\begin{array}{c} \ln\;\dot{\varepsilon }\;={\ln A}_{2}+\beta \sigma -\frac{Q}{RT} \end{array}$$

**Figure 6:**
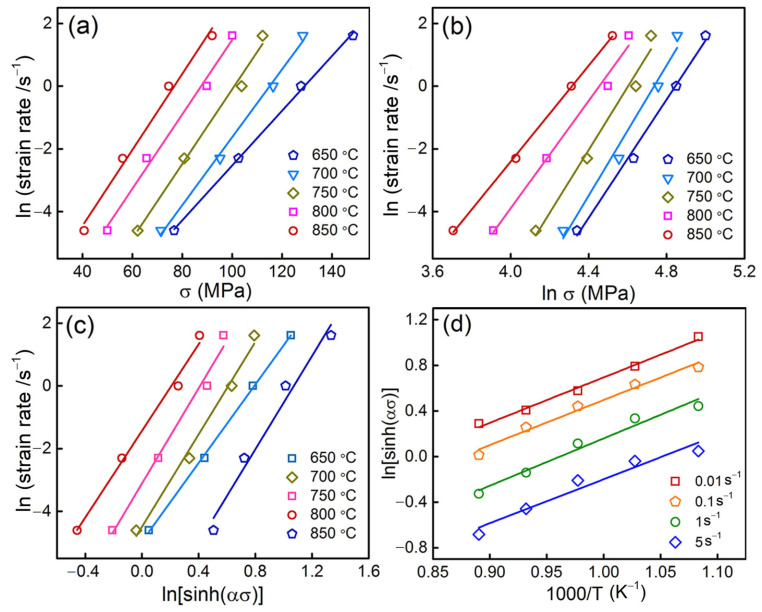
Relationships between (**a**) $\ln\dot{\varepsilon }$ − σ; (**b**) $\ln\dot{\varepsilon }$ − lnσ; (**c**) $\ln\;\dot{\varepsilon }\;$ − $\ln\sin h\left(\alpha \sigma \right)$, and (**d**) $\ln\;\dot{\varepsilon }\;$ − 1000/*T*.

*β* and *n*_1_ were calculated to *β* = 0.1154 MPa^−1^ and 9.23 MPa^−1^ by the linear relationships of σ*−*$\ln\;\dot{\varepsilon }\;$ (Figure 6a) and lnσ*−*$\ln\;\dot{\varepsilon }\;$ (Figure 6b), respectively. Thus, the *α* value of 0.01194 MPa^−1^ can be obtained according to *α* = *β*/*n*_1_. By taking the logarithm of both sides of Equation (3), it yields:

(7) $$\begin{array}{c} \ln\left[\sin h\left(\alpha \sigma \right)\right]=\frac{1}{n}\ln\;\dot{\varepsilon }\;+\frac{Q}{nR}\left(\frac{1}{T}\right)-\frac{1}{n}\ln A \end{array}$$

Assuming the strain rate is constant, the above equation can be rewritten as follows:

(8) $$\begin{array}{c} Q=R{\left[\frac{\partial \left(\ln\;\dot{\varepsilon }\;\right)}{\partial \ln\left[\sin h\left(\alpha \sigma \right)\right]}\right]}_{T}{\left[\frac{\partial \ln\left[\sin h\left(\alpha \sigma \right)\right]}{\partial \left(1/T\right)}\right]}_{\;\dot{\varepsilon }\;}=RnS \end{array}$$

Figure 6c,d shows the linear relationships of $\ln\dot{\varepsilon }-\ln\left[\sin h\left(\alpha \sigma \right)\right]$ and $\ln\left[\sin h\left(\alpha \sigma \right)\right]-1000/T$, respectively, from which the strain index *n* (7.16) and *S* (3.98) can be achieved by calculating the slopes of the fitting lines. The value of *Q* for the prepared ZnSnO_3_/Cu composite is 237.05 kJ/mol according to *Q* = *RnS*.

The Zener–Hollomon parameter *Z* was analyzed in order to probe the effects of deformation conditions on the thermal processing performance of the materials. By taking the natural logarithm of both sides of Equation (4), the relationship between lnZ and $\ln\left[\sin h\alpha \sigma \right]$ for the ZnSnO_3_/Cu composite can be written as:

(9) $$\begin{array}{c} \ln Z=\ln A+n\ln\left[\sin h\left(\alpha \sigma \right)\right] \end{array}$$

**Figure 7:**
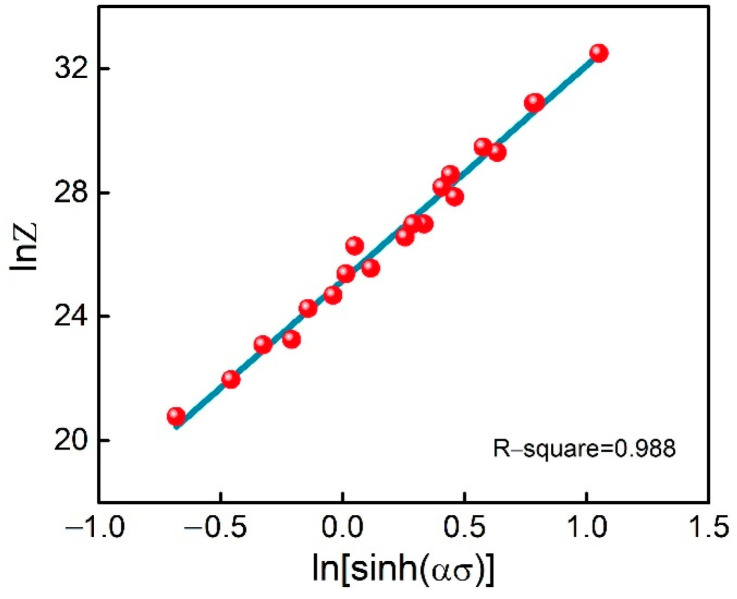
Relationship between lnZ and $\ln\sin h\left(\alpha \sigma \right)$ of ZnSnO_3_/Cu composites.

Figure 7 depicts the above relationship, yielding the value *A* = 8.56 × 10^10^. Thus, the constitutive equation of ZnSnO_3_/Cu composites was determined as:

(10) $$\begin{array}{c} \;\dot{\varepsilon }\;={8.56\times 10}^{10}{\left[\sin h\left(0.011947\sigma \right)\right]}^{7.156}\exp\left(-\frac{237.05}{RT}\right) \end{array}$$

The *Q* value reflects the energy barrier for the hot diffusion of atoms [39]. Compared with TiC/Cu (269.059 kJ/mol) [40], SiC/Cu (320.79 kJ/mol) [41], and diamond/Cu (238 kJ/mol) [42], other Cu-based alloys (300–500 kJ/mol) [43,44,45,46], ZnSnO_3_/Cu composites possess a lower thermal activation energy (237.05 kJ/mol). It is accepted that lower activation energy yields easier hot deformation of the composites. The results signify that the dislocation movement and DRX of ZnSnO_3_/Cu were more prone to occur in the composite. Additionally, it can also be deduced that trace Zr (0.56 wt.%) and La (0.005 wt.%) elements in the composite played important roles in the refinement of grain size according to the previous study [47,48], which was supposed to accelerate the dynamic recrystallization of the composites. These aspects contribute to good hot workability of the designed composites.

### 3.4. Processing Maps

Processing maps are utilized to optimize hot deformation processing based on which the optimal processing parameters can be determined for industrial production. According to the model developed by Prasad et al. [49], the deformation workpiece was considered as an energy dissipater [50,51]. Under hot deformation, the total dissipated energy, including complementary parts of *G* and *J*, can be written as:

(11) $$\begin{array}{c} P=G+J=\sigma \;\dot{\varepsilon }\;=\overset{\dot{\varepsilon }\;}{\underset{0}{\int }}\sigma d\;\dot{\varepsilon }\;+\overset{\sigma }{\underset{0}{\int }}\;\dot{\varepsilon }\;d\sigma \end{array}$$

The strain rate sensitivity parameter (*m*) determines the *G* and *J*, which can be described as:

(12) $$\begin{array}{c} m=\frac{dJ}{dG}={\left[\frac{\partial \left(\ln\sigma \right)}{\partial \left(\ln\;\dot{\varepsilon }\;\right)}\right]}_{\varepsilon ,\;T} \end{array}$$

The power dissipation capacity of the composites can be appraised by the energy dissipation (η), and for linear dissipation process, where m=1 and ${J=J}_{\max}=\frac{\;\sigma \dot{\varepsilon }\;}{2}=\frac{P}{2}$, η can be written as:

(13) $$\begin{array}{c} \eta =\frac{J}{J_{\max}}=\frac{2m}{m\;+\;1} \end{array}$$

The extremum principles of irreversible thermodynamics as applied for a large plastic flow body are utilized to determine flow instability, which can be expressed as:

(14) $$\begin{array}{c} \xi \left(\;\dot{\varepsilon }\;\right)=\frac{\partial \ln\left(\frac{m}{m\;+\;1}\right)}{\partial \ln\;\dot{\varepsilon }\;}+m<0 \end{array}$$

Therefore, the variation of $\;\xi \left(\dot{\varepsilon }\right)$ with T and $\;\dot{\varepsilon }\;$ can be directly reflected in the instability map, according to which the regions with negative $\;\xi \left(\dot{\varepsilon }\right)$ values can be delineated.

**Figure 8:**
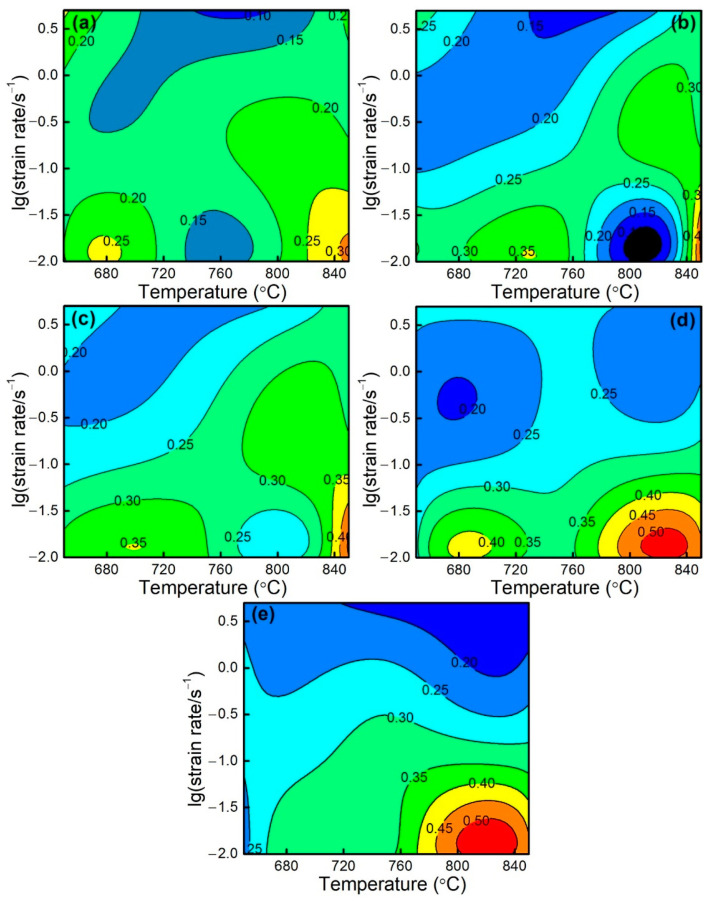
Processing maps of ZnSnO_3_/Cu composites at a true strain of (**a**) 0.1, (**b**) 0.5, (**c**) 0.7, (**d**) 0.9, and (**e**) 0.95.

**Figure 9:**
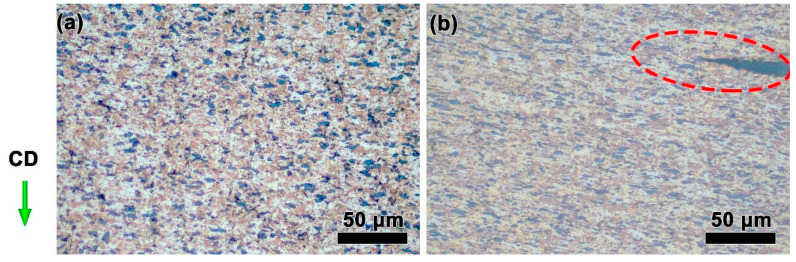
Microstructures of the ZnSnO_3_/Cu composites at 800 °C with different strain rates: (**a**) 0.01 s^−1^, (**b**) 1 s^−1^. CD presents the compression direction of the sample. The red dotted circle in (**b**) indicates crack.

Figure 8 illustrates the processing maps of ZnSnO_3_/Cu at a strain of 0.1–0.95. The efficiency of power dissipation is indicated by blue–red color coding [0.1, 0.5], and the unstable region is represented by blue with low values. When deforming at a low strain of 0.1–0.5 (Figure 8a,b), low efficiency of power dissipation occurs. This result demonstrates that defects, including crack and adiabatic shear band, could generate in the deformed ZnSnO_3_/Cu composites, resulting in inferior workability of the materials. Thus, these domains with corresponding parameters should be avoided during thermal processing. Simultaneously, the efficiency of power dissipation increases, and the stable region significantly enlarges with the rising temperature or the reduction of the strain rate, especially when the true strain exceeds 0.9 (Figure 8d). This indicates that the composites deformed under these circumstances exhibit excellent workability [52]. As shown in Figure 8e, a higher efficiency of power dissipation (>45%) appears in the region of 790–850 °C and 0.01–0.04 s^−1^. To confirm the processing map results, hot deformation tests were performed at 0.01 and 1 s^−1^, respectively, and the microstructure is shown in Figure 9. It can be observed from Figure 9a that, at a higher efficiency of power dissipation (800 °C and 0.01 s^−1^), the composites deformed with good workability. Upon deviating from this domain by improving the strain rate to 1 s^−1^, a crack indicated by red-dashed circle occurred in Figure 9b, which was unfavorable for hot working. Thus, the obtained domain provides optimal processing parameters for improving the thermal processing performance of materials and obtaining high-quality products during practical applications.

## 4. Conclusions

In this work, Cu-based composites reinforced with ZnSnO_3_ ternary oxide were designed, and the hot deformation behavior of the composites was evaluated at 650–850 °C and 0.01–5 s^−1^ strain rates. It was found that, when increasing the temperature and decreasing the strain rate, the high-temperature flow stress and the corresponding peak stress of ZnSnO_3_/Cu composites decreases. Dynamic recovery was found to be the primary softening mechanism at low temperature and high strain rate, while dynamic recrystallization occurred with increasing temperature and decreasing strain rate. The activation energy was calculated as 237.05 kJ/mol, and the constitutive equation can be expressed as follows:$\;\;\dot{\varepsilon }\;={8.56\;\times \;10}^{10}{\left[\sin h\left(0.11947\sigma \right)\right]}^{7.156}\exp\left(-\frac{237.05}{RT}\right)$. Based on the established processing map, the optimum domain with an efficiency of power dissipation of 45% for the hot working of ZnSnO_3_/Cu composites occurred at 790–850 °C and 0.01–0.04 s^−1^ strain rate, which is beneficial for the plastic deformation of high-quality products.

## Data Availability

The data presented in this study are available on request from the corresponding author.
